# Use of Probiotics for Preventing Necrotizing Enterocolitis in Preterm Infants: A Survey of Current Practices Among Indian Neonatologists

**DOI:** 10.7759/cureus.73923

**Published:** 2024-11-18

**Authors:** Kiran More, Anil Hanumantharaju, Astha Amrit, Somashekhar M Nimbalkar, Sanjay Patole

**Affiliations:** 1 Neonatology, MRR Children's Hospital, Thane, IND; 2 Neonatology, Pramukhswami Medical College, Bhaikaka University, Karamsad, IND; 3 Neonatology, Mount Sinai Hospital, Toronto, CAN; 4 Central Research Services, Bhaikaka University, Karamsad, IND; 5 Pediatrics, Pramukhswami Medical College, Karamsad, IND; 6 Neonatology, King Edward Memorial Hospital, Perth, AUS

**Keywords:** india, necrotizing enterocolitis (nec), preterm premature, probiotics and microbiome, survey research

## Abstract

Objective: Probiotics are known to reduce the risk of necrotizing enterocolitis (NEC≥ Stage II) significantly, as well as all-cause mortality, late-onset sepsis (LOS), and feeding intolerance in preterm infants. Probiotics have been reported to have comparable benefits in high- and low-middle-income countries (LMICs). We aimed to assess the current practices of neonatologists in India for using probiotics in preterm infants.

Material and methods: A questionnaire created using Survey Monkey's web-based tool was sent to neonatologists in India. Survey forms automatically converted responses into Excel files (Microsoft® Corp., Redmond, WA). Data were analyzed using SPSS (IBM Corp., Armonk, NY).

Results: A total of 615 responses were received from various neonatal intensive care units (NICUs) in India (Level I: 43 (7%), II: 124 (20.8%), III: 448 (72.8%)). Around 431 (70%) of the units had either National Neonatology Forum (NNF) accreditation or IAP fellowships or were affiliated with private or government medical colleges. The remaining 184 (30%) were in private setups. Routine probiotic supplementation (RPS) was provided in 241 (39.1%) of the responding units; 179 (48%) quoted inadequate evidence as the reason for not providing RPS, 125 (33.43%) quoted difficulty in sourcing safe and effective products, whereas others were concerned about adverse effects. Most centers provided RPS for preterm infants <32 weeks and 1500 g at birth. The clinical practice was influenced by the judgment of the attending clinician. Significant variation was noticed in the protocol for RPS.

Conclusion: Findings of the survey suggest that approximately 39% of the participating neonatologists in India currently offer RPS for preterm infants. A significant variation exists in the selection of probiotic strains, products, dose, and duration of supplementation. Despite limitations, our findings are useful in guiding clinical practice and further research to optimize the safety and efficacy of RPS for preterm infants.

## Introduction

Survival of very preterm (gestation <32 weeks) infants has increased significantly in the surfactant era. This population of infants is at high risk for mortality and morbidities such as late-onset sepsis (LOS), necrotizing enterocolitis (NEC), and feeding intolerance [1]. The risk of such adverse outcomes is particularly high in extremely preterm (gestation <28 weeks) infants.

Probiotics are live microorganisms that, when administered adequately, provide numerous benefits for the host [2]. Systematic reviews of randomized controlled trials (RCTs) and non-RCTs have shown that probiotic supplementation significantly reduces the risk of all-cause mortality, NEC ≥ Stage II, LOS, and feeding intolerance in preterm, very low birth weight (VLBW) infants [3]. Probiotics' mechanisms of benefits in this context include their anti-inflammatory and immunomodulatory action, as well as their ability to enhance the gut barrier and modulate the gut microbiota for host benefits [4,5].

The benefits of probiotics have been reported to be comparable between high- and low-middle-income countries (LMICs) [6]. A systematic review of probiotic RCTs in preterm infants in India supports these findings [7]. Despite the significant evidence from RCTs (total participants = 15,700) [8] and non-RCTs (total participants = 77,000) [9], the uptake of probiotic supplementation for preterm infants has been relatively slow for various reasons, including the uncertainty about optimal probiotic strain/s, dose and duration of supplementation, difficulties in accessing high-quality probiotic products, inadequate data on safety and efficacy of probiotics in extremely preterm infants, concerns about probiotic sepsis, and the potential for development of antibiotic resistance [10].

Given the potential of probiotic supplementation to improve the outcomes of this high-risk cohort of preterm infants and the uncertainties about this intervention, it is important to know the current practice in this field in India. We aimed to conduct a national survey to assess the current practices of Indian neonatologists regarding probiotic supplementation for preterm infants.

## Materials and methods

A self-administered questionnaire was created on a web-based tool, Survey Monkey, and responses were automatically collected on the portal. Institutional ethics committee approval was obtained. A list of physicians registered with the National Neonatal Forum (NNF) of India was requested from the official NNF portal. A similar list was also obtained from the Indian Academy of Paediatrics (IAP) database. Before the participants agreed, we emailed them the brief content and purpose of the survey to make an informed judgment on whether to participate or not. The survey link was sent to consenting neonatologists via email, the WhatsApp platform, and various common forums. Four investigators individually sent the message with a gap of two to three days in between to maximize the response.

The survey questions were designed to assess various aspects of clinical practice for probiotic supplementation for preterm infants. The questions covered issues such as the eligibility criteria based on birth weight and gestation, type of probiotic strains and products, probiotic dose, duration, and protocol for preparing and administering the probiotic to the infants and for storing the product, the level of the neonatal intensive care unit (NICU), and data on associated complications such as probiotic sepsis. Information was also collected from the clinicians about their opinions, behavior, or knowledge related to probiotics (appendices). 

Each response was linked to a unique identifier for individual responders to avoid duplication of responses. The survey form could be submitted only when completed to prevent incomplete answers; few questions were mandatory. All questions were proofread and pilot-tested for accuracy by the co-authors before loading the questionnaire on the Survey Monkey website. Furthermore, we administered the survey questionnaire to 10 volunteer neonatologists to ensure consistency of responses. 

Confidentiality of the respondents was maintained at all stages, and the survey responses were accessible only to authorized authors.

Statistical analysis

Survey forms automatically converted every questionnaire into Excel files (Microsoft, Seattle, WA). Every questionnaire was carefully checked for inconsistencies throughout this conversion process. Continuous variables were presented as means (SD), and categorical variables were presented as proportions. SPSS v 20 (SPSS Inc., Chicago, Illinois) was used for the statistical analysis.

## Results

A total of 615 out of the ~850 centers across India responded to the survey after accounting for multiple entries. Four hundred thirty-one (70%) of the responding centers had either NNF accreditation or Fellowship of the IAP/NNF fellowships or were affiliated with private or government medical colleges. The remaining 184 (30%) centers were from private setups. Table 1 describes the characteristics of the NICUs. The majority of the centers provided level 3 care and offered both intramural and extramural deliveries. Average annual admissions of 1072 newborns were seen across the centers, and about 365 (34%) of admissions belonged to gestational age of <32 weeks.

**Table 1:** Centers participating in the survey Data has been represented as frequency N (%).

Characteristics	Frequency (%)
Level of care	
Level 1	43 (7%)
Level 2	124 (20.8%)
Level 3	448 (72.8%)
Type of admissions	
Intramural and extramural	532 (86.5%)
Only extramural	83 (13.5%)
Annual admissions (average 1072)	
<32 weeks	365 (34%)
>32 weeks	707 (66%)

The responses to the questions about unit policy for routine probiotic supplementation (RPS) are summarized in Table 2. About 241 (39.1%) centers had provisions for RPS, and only 96 (39.83%) of those 241 centers had written policies for RPS. One hundred and seventy-nine (47.8%) respondents cited inadequate current evidence as the most common reason for not implementing RPS in 374 centers. Most of the responding centers started providing RPS for preterm infants in 2018-2019. One of the units started providing RPS as early as 2011.

**Table 2:** Provision of routine probiotic supplementation in NICU RPS: routine probiotic supplementation. Data has been represented as frequency N (%).

Parameters	Responses	Frequency (%)
Provision of RPS*	Yes	241 (39.1%)
	No	374 (60.9%)
Written policy for RPS among those who have provision (241)	Yes	96 (39.83%)
	No	145 (60.17%)
Reasons for not providing RPS (374)	Inadequate current evidence	179 (47.86%)
	Difficulty in sourcing a safe and effective product	125 (33.42%)
	Concerns about adverse effects	26 (6.95%)
	Difficulty in handling regulatory processes	19 (5.08%)
	Others	25 (6.68%)

Plan for RPS among centers without RPS

A total of 161 (43%) units are planned to provide RPS in the near future. Sixty-three (16.8%) did not wish to provide RPS anytime soon, and 150 (40.1%) were uncertain about providing it in the future.

The features of RPS

Gestation Age at Starting RPS

The majority of the responding units provided RPS for infants born <32 weeks, and others (in decreasing frequency) provided it for infants <34, <30, and <28 weeks. The majority provided RPS for infants with birth weight <1500 g, and others provided it for those <1200 or <2000 g. Most centers started RPS on reaching a feeding volume of 15-20 ml/kg/day, and few started it before the initiation of feeds. The majority of centers continued RPS till 34 weeks of corrected gestational age and reaching full feeds, with few continuing RPS till 40 weeks or discharge. Most units followed a twice-daily regimen, and others followed a thrice-daily regimen. A dose of 1.25 × 109 CFU to 2.5 × 109 colony-forming units per day was used in most centers. For reconstitution, most centers used breastmilk, and others used sterile/distilled water. Most probiotic formulations were in powder form. Other forms included drops, syrup, and capsules. The shelf life of probiotic products ranged from six months to two years for most of them, and all units stored the probiotic in a cool and dry place.

The characteristics of commonly used probiotic products are shown in Table 3. The majority of these contained *Lactobacillus acidophilus*, *L. rhamnosus*, *Bifidobacterium longum*, *B. breve*, *Saccharomyces boulardii*, and *Bacillus clausii*.

**Table 3:** Name and composition of probiotics used in the responding centers

Probiotic brand	Composition	Number of centers (n=111)
Darolac	Lactobacillus acidophilus, Lactobacillus rhamnosus, Bifidobacterium longum, Saccharomyces boulardii	56
Rescunate	Bifidobacterium breve M-16v	27
Superflora drops	Lactobacillus rhamnosus	12
Econorm	Saccharomyces boulardii CNCMI-745	5
Sporolac	Lactobacillus sporogenes, Bacillus Clausii, Bacillus Subtilis	2
Bifilac	Lactobacillus, Streptococcus faecallis, Bacillus mesentricus, Clostridium butyricum	2
Pro GG	Lactobacillus rhamnosus GG	2
Vizylac GG	Lactobacillus rhamnosus GG ATCC 53103	2
Enterogermina	Bacillus Clausii	2
Sporonorm	Lactobacillus, Saccharomyces boulardii	1

Strain Genomics

Most neonatologists were unsure if the data on the full genome of the probiotic strain were available and did not have information on the antibiotic sensitivity of the probiotic strain, 283 (46%). The rest thought such data were unavailable, and only 74 (12%) were aware of such information.

Adverse Effects

Among the 241 neonatologists who practiced RPS, the overwhelming majority, 231 (96%), did not report any significant adverse events related to RPS. Few reported vomiting and abdominal distension associated with RPS. As for probiotic sepsis, 147 (61%) of clinicians who practiced RPS were unsure whether the blood culture methods available at their center could detect sepsis caused by the administered probiotic strains. The majority were willing to assist in the standardization of RPS in their region and contribute "minimum" data to a probiotic registry.

Handling Probiotic Stock

Probiotic products were commonly stored at the bedside and within the NICU facility. However, only some centers obtained the probiotics daily from the pharmacy instead of storing the stock in the NICU. In most centers, nursing and pharmacy staff monitored the stock. Approval from the institutional ethics committee or drug committee was not required for RPS in 170 (70.53%) of centers that practiced RPS.

## Discussion

Our survey findings offer valuable insights into the current practice of RPS for preterm infants across many NICUs in India. To the best of our knowledge, this is perhaps the first such survey conducted in India. A total of 615 replies were received. Knowing the precise response rate is difficult because we used WhatsApp, an open platform. The reply from 431 NICUs with either NNF accreditation or NNF/IAP fellowships represents a ~ 50% response rate, considering that there are ~850 such NICUs in India. This response rate can be considered low but is similar to that observed in a recent international survey on COVID-19. It is important to note that the analysis of answers in the aforementioned survey was conducted without a specified denominator [11]. 

RPS was practiced in 241 (39.19%) of the NICUs in our survey. This finding is noteworthy, considering the significant difference in the practice of RPS reported in other international surveys. A survey conducted across 25 NICUs in Canada [12], showed that RPS was practiced in 72% of NICUs. The results of another national survey show that RPS was practiced in ~68% of the NICUs in Australia and New Zealand [13]. A survey conducted in 2021 by the Vermont Oxford Network showed that the rate of RPS was only ~5.7% for VLBW neonates and 8.3% for infants at all gestational ages [14]. Findings of another survey from the USA in 2015 showed that only 8.8% (44/500) of NICUs were providing RPS, and probiotics were used on a selective basis in 5.2% (26/500) of the NICUs [15]. However, the results of a cohort study (data up to 2019) have shown a significant increase (to 16.5%) in the use of RPS in the USA [16]. The increase in adoption of RPS was correlated with reduced risk of NEC, without any effect on other outcomes. A survey from England has reported that ~17% of the NICUs used RPS [17]. Another survey from Spain showed that donor milk and breastfeeding promotion were widely used (87%), whereas probiotics were used less frequently (23%) in their NICUs.

Various studies have shown mixed results on the effectiveness of probiotics in reducing NEC in high-risk preterm infants, with no pharmaceutical-grade product available in the U.S. and long-term safety still uncertain. While the American Academy of Pediatrics does not universally recommend probiotics for preterm infants, especially those under 1000 g, centers that choose to use them should have informed consent, follow local guidelines, and be aware of the lack of regulatory standards and potential contamination [18].

Our results show that "inadequate evidence" supporting the intervention is a significant barrier to adopting RPS in Indian NICUs. The influence of the Cochrane systematic review is important in this context [19]. Other barriers against adopting RPS for preterm infants include the difficulty in accessing a safe, effective probiotic product, concerns about probiotic sepsis, antibiotic resistance, and difficulties handling the know-how for the process of regulatory approvals. The regulatory guidelines for probiotics in India are framed by the Indian Council of Medical Research (ICMR) in association with the Department of Biotechnology (DBT). ICMR also envisaged the formation of a special regulatory body, 'The Foods Safety and Standards Authority of India,' to monitor all food-relevant issues [20].

The fact that only a few of the responding units had a unit guideline/policy for providing RPS for preterm infants is important as it reflects uncertainty about the safety and efficacy of probiotics in this high-risk population. Many of the responding neonatologists shared their hesitation about using RPS for extremely preterm (gestation <28 weeks) or extremely low birth weight (ELBW: <1000 g) despite the supporting evidence [21]. The postnatal age at commencing and the dose and duration of probiotics for routine prophylaxis were in accordance with the various recommendations [22-25].

As for probiotic products, our survey showed that Darolac™ and Rescunate™ were commonly used in the responding NICUs. Probiotic species, including *Lactobacillus acidophilus*, *Lactobacillus rhamnosus*, *Bifidobacterium longum*, *Bifidobacterium breve*, *Saccharomyces boulardii*, and *Bacillus clausii* were found in the majority of the probiotic products used in the NIUCs participating in our survey. Barring a few exceptions (e.g., Saccharomyces), the probiotic species used for RPS were in accordance with the recommendations of the European Society for Paediatric Gastroenterology, Hepatology, and Nutrition (ESPGHAN).

Importantly, our survey results show the lack of facilities for on-site laboratory surveillance to monitor the risk of probiotic sepsis. Several investigations have shown that the microbial content of a probiotic product may differ from what is stated on the product label [26].

The results of a systematic review show that probiotics have the potential to significantly improve survival and reduce morbidities such as NEC and LOS in preterm infants in low-middle-income countries (LMICs) such as India [6]. Furthermore, results of a systematic review and meta-analysis of probiotic RCTs (n=9, 1514 participants) conducted in India show that probiotic supplementation decreased the incidence of NEC, LOS, and all-cause mortality in preterm infants [6].

Our survey findings suggest an urgent need to address the various concerns that delay the widespread adoption of RPS in India. The guidelines provided by Deshpande et al. [26] and recommendations by other expert bodies [8] are important in this context.

The findings of the large NIH-funded study by Panigrahi et al. [27] and the currently ongoing large multi-center trial (ProSPoNS) in India will help guide the efforts to optimize the intake of RPS for preterm infants [28]. It is important to note that the World Health Organization (WHO) guideline development group has recently focused on systematic reviews that addressed 24 research questions for global healthcare interventions for preterm and low birth weight infants. Based on the evidence, the expert group has included probiotic supplementation as an intervention to improve the outcomes of preterm LBW infants [29].

The lack of clarity in the denominator for estimating the precise response rate can be considered an important limitation of our survey. However, we believe that our findings do provide meaningful data to guide further research and clinical practice in this field.

The limitations and strengths of our survey need to be acknowledged. To the best of our knowledge, this is the first prospective self-administered survey of the current practices among Indian neonatologists on probiotic use in preterm infants. Our findings provide insight into the prevalence of RPS in the participating centers. They also provide useful information on the variation in practice on important aspects of RPS, including the selection of probiotic strains and products, storage, as well as the dose and the duration of supplementation. 

The limitations of our findings include the unclear denominator to accurately assess the response rate, a crucial factor in judging the validity of the findings of such surveys. It is difficult to know how many neonatologists viewed the questionnaire and opted not to respond. It is also possible that the "online" format of the questionnaire may not have been an attractive option for busy practitioners. Despite the limitations, we believe they will help guide research and practice in this field. Our results will also help the national regulatory agency in developing guidelines for probiotic supplementation for preterm infants to optimize the uptake of this intervention.

## Conclusions

In summary, our findings suggest that 39.1% of the neonatologists in India who participated in our survey currently offer RPS for preterm infants. Significant variation exists in the selection of probiotic strains, products, dose, and duration of supplementation. Despite their limitations, our findings are helpful in guiding clinical practice and further research to optimize the safety and efficacy of RPS for preterm infants.

## Appendices

**Table 4:** Survey questions on survey platform

SL NO.	Questions	Options
1.	Level of NICU care	Level 1/level 2/level3
2.	Number of NICU beds	
3.	Admission rates per year (<32 weeks and >32 weeks)	
4.	Contact details Physicians name NICU name Country Email address Phone number	
5.	Does your unit provide routine probiotic supplementation (RPS) for preterm infants	Yes/no
6.	If ‘no’, please provide reasons (can select as many options as needed)	Believe that current evidence is inadequate. Difficulty in handling regulatory process. Difficulty in sourcing safe and effective probiotic product. Concerns about adverse effects. Others (please specify).
7.	Are you planning to start RPS in near future	Yes/no/not sure
8.	Is there a protocol for RPS in your unit	Yes/no
9.	Date when RPS was started	
10.	Please email a copy of the protocol if possible (kmore@sidra.org)	
11.	Please specify birth weight and gestational age in weeks considered for commencing RPS in your unit	
12.	Please select what minimal feeding criteria you follow before commencing	With non-nutritive sucking. Feed volume <20 ml/kg/day. After reaching certain feed (specify amount). Before starting feeds. No specific criteria.
13.	Postnatal age at stopping RPS	
14.	Name of the product	
15.	Product type	Powder/capsule/syrup/tablet
16.	Probiotic strain details	
17.	Frequency of administration	Once/twice/thrice/four times per day
18.	Daily dose (CFU per day)	
19.	Product used for reconstitution	Breast milk/sterile water/others
20.	Method of procuring product	Approval from hospital ethics board/Approval from drug committee/Approval from hospital pharmacy/Other approval required (specify)/None required.
21.	Where do you store the stock of probiotic	Within NICU facility/Bedside/In pharmacy/Other.
22.	How do you maintain the probiotic stock (select as many)	Dedicated person monitoring stock/Safe disposal of expired stock/Maintaining the log of product.
23.	Methods of monitoring RPS related adverse effects (select as many)	Clinical monitoring/Blood culture/ Others (specify).
24.	If you have any idea about genome of probiotic product?	Yes/no
25.	Antibiotic sensitivity pattern of NICU	
26.	Method of detecting probiotic stains in culture	Bactec blood/Bactalert/Conventional method.
27.	Shelf life of probiotic product	
28.	Storage conditions of probiotic product	Room temperature/Cold storage/Others (specify).
29.	Have you experienced any adverse effects related to RPS?	Yes/no. If yes specify.
30.	Currently used methods of routine blood culture	Bactec/Bactalert/Conventional.
31.	Can this method detect sepsis caused by probiotic strain?	Yes/no
32.	Any publication/presentations on probiotics from your unit?	Yes/no
33.	Will you be willing to contribute in standardizing RPS in the region to guide clinical practice?	Yes/no
34.	Would your unit be willing to contribute to a probiotic registry for minimum data on outcomes to discharge/death on neonates receiving RPS?	Yes/no
